# Against Explanatory Minimalism in Psychiatry

**DOI:** 10.3389/fpsyt.2015.00171

**Published:** 2015-12-07

**Authors:** Tim Thornton

**Affiliations:** ^1^College of Health and Wellbeing, University of Central Lancashire, Preston, UK

**Keywords:** Campbell, levels of explanation, intentionality, mechanism, rationality, Wittgenstein

## Abstract

The idea that psychiatry contains, in principle, a series of levels of explanation has been criticized not only as empirically false but also, by Campbell, as unintelligible because it presupposes a discredited pre-Humean view of causation. Campbell’s criticism is based on an interventionist-inspired denial that mechanisms and rational connections underpin physical and mental causation, respectively, and hence underpin levels of explanation. These claims echo some superficially similar remarks in Wittgenstein’s *Zettel*. But attention to the context of Wittgenstein’s remarks suggests a reason to reject explanatory minimalism in psychiatry and reinstate a Wittgensteinian notion of levels of explanation. Only in a context broader than the one provided by interventionism is that the ascription of propositional attitudes, even in the puzzling case of delusions, justified. Such a view, informed by Wittgenstein, can reconcile the idea that the ascription mental phenomena presupposes a particular level of explanation with the rejection of an *a priori* claim about its connection to a neurological level of explanation.

## Introduction

Psychiatry deals with phenomena that range between large-scale higher order social phenomena (e.g., poverty, cultural norms), person level phenomena (e.g., trauma, symptoms such as delusions and syndromes such as depression), and the sub-personal level phenomena (e.g., genes, neurones). It advances explanations that invoke a variety of factors across the scale and both proximal and distal. It is tempting to think that this apparent heterogeneity could be simplified, in principle at least, by fitting it into a picture of different levels of explanation – whether ontological or epistemological – which relate together in some general ways.

The actual applicability of this picture to present psychiatry has been contested (1). Typically, psychiatry trades across levels. This paper, however, describes a principled attack on the very idea of levels of explanation in favor of a form of explanatory minimalism. Put roughly, causal explanation can trade across putative levels because causation is brute and answers to no *a priori* conditions of intelligibility. That being so, the very idea of a “level of explanation” – which depends on such *a priori* assumptions about intelligibility – is undermined. Or so John Campbell argues in a number of papers (2–4).

Having set out Campbell’s argument for explanatory minimalism for psychiatry, I compare it to some similar sounding remarks from Wittgenstein’s collection *Zettel* (5). Like Campbell, Wittgenstein denies the necessity for mechanisms to mediate (apparently) causal connections and also denies the assumption that rational connections between mental phenomena need be mediated by underlying neurological mechanisms. But Wittgenstein’s remarks are aimed at undermining mechanistic accounts of the intentional directedness of mental states, not at denying that there is a characteristic level of explanation for mental phenomena. The problem lies not with the idea of levels of explanation but with an unwarranted metaphysical assumption about how they relate.

The final section builds on Wittgenstein’s account of the normative connections between mental phenomena and argues that Campbell’s explanatory minimalism is insufficient for psychiatry because it provides no account of what constitutes states as mental states, which plays an important role in psychiatric explanation.

## Background: Levels of Explanation

There are two dominant approaches to the idea of levels of explanation: ontological and epistemological. Both attempt to shed light on the idea of levels of explanation by characterizing the differences between the levels and also the constraining relations between them.

The ontological view is part of a traditional reductionist picture of the world. On this picture, sciences of the mind, such as psychiatry and psychology, can in principle be reduced to biology (which might be construed as physiology or evolutionary biology), biology to chemistry, and chemistry to physics. Oppenheim and Putnam expressed this view in their classic 1958 paper “Unity of science as working hypothesis.”

It is not absurd to suppose that psychological laws may eventually be explained in terms of the behaviour of individual neurons in the brain; that the behaviour of individual cells – including neurons – may eventually be explained in terms of their biochemical constitution; and that the behaviour of molecules – including the macromolecules that make up living cells – may eventually be explained in terms of atomic physics. If this is achieved, then psychological laws will have, *in principle*, been reduced to laws of atomic physics… [Ref. (6): p. 407]

Oppenheim and Putnam go on to argue that the unity of science is served by “microreductions.” These are reductions in which:

The objects in the universe of discourse of [the reduced science or theory] are wholes which possess a decomposition into proper parts all of which belong to the universe of discourse of [the reducing science or theory] [ibid: 407].

In fact, they argue more strongly that microreduction is the *only* method seriously available for the unity of science [ibid: 408]. They then go on to explore the consequences of this view by examining the preconditions for successfully attaining unity via microreduction. Since microreduction is construed as the only serious possibility for the unity of science, and since its success rests on a number of other things being the case, the goal of unification has a number of presuppositions:

1. There must be several levels.
2. The number of levels must be finite.
3. There must be a unique lowest level …
4. Anything of any level except the lowest must possess a decomposition into things belonging to the next lowest level … [ibid: 409].

This list suggests the following view of nature and the constraining relations between levels of explanation. The world is made up of basic building blocks or atoms, which display regularities that can be described in the law statements of the most basic science. The basic atoms also combine to form larger structures that display characteristic regularities of their own. These can in turn be codified in the law statements of higher level sciences. But the higher level regularities do not emerge out of nothing. They can be explained as the consequences of the more basic patterns of behavior of atoms. So, the structure of the world and the structure of science can be seen as two isomorphic hierarchies of levels.

The picture suggests three interrelated mutually reinforcing views of the levels of explanation. First, they correspond to different disciplines within science. Second, higher levels contain objects that are constituted from lower level objects. Third, higher level objects are larger than lower level objects. These views fit together on the assumptions that different sciences study objects at different scales and that objects only interact with other objects at the same level. However, these assumptions have been criticized (7).

There is, however, another and quite different approach to levels of explanation, which has been influential. It is based not on the size and composition but rather degree of abstraction to higher order causal processes. This is Marr’s threefold epistemological distinction between:

*Computational theory*: What is the goal of the computation, why is it appropriate, and what is the logic of the strategy by which it can be carried out?

*Representation and algorithm*: How can this computational theory be implemented? In particular, what is the representation for the input and output, and what is the algorithm for the transformation?

*Hardware Implementation*: How can the representation and algorithm be realized physically? [Ref. (8): p. 25]

This hierarchy does not concern different ontological levels but rather different ways of understanding the same ontology. The highest, most abstract level concerns the function of a system. It might be carried out by a variety of different algorithms at the middle level. Finally, the same algorithm might be realized in different physical ways at the lowest level. Thus, the higher levels are multiply realizable by lower levels. Determining the computational level is a matter of determining the goals of a system independently of its physical or neurological properties.

Although Marr’s epistemic approach seems appropriate for its original application to vision, where the goals of the system can be theorized about independent of algorithm and physiological realization, it is less clear that it applies to psychiatry. As Dominic Murphy argues, actual practice in psychiatry is to determine the functions of systems in part with a view of what the lower level physiology could sustain. “[O]ur understanding of realisation feeds back into and constrains our understanding of the abstract demands of cognition” [Ref. (1): p. 105].

Neither, however, does the ontological view of levels of explanation fit psychiatry because “causes described in genetic vocabulary will be related to effects described in terms of behavioural tests, for example, and generalisations will cross levels” [ibid: 108]. Thus, according to Murphy, there are different systems operating at different levels, unlike the epistemic view, but the different levels interact, unlike the ontological view.

Murphy’s argument starts from a relaxed approach to the nature of levels – “I have little to say about what levels of explanation actually are” [ibid: 103] – and then argues that they do not apply to psychiatry. Whatever they are, psychiatric explanation typically crosses them. Such an argument, however, leaves open the response that it merely reflects the current imperfect state of psychiatry. It is tempting to think that Oppenheim and Putnam’s picture reflects how reality must be structured even if, for contingent reasons, causal generalizations can link different levels. Equally, the fact that knowledge of physical realization informs more abstract theories of function need not conflict with the idea that there are, in principle, different levels of abstraction applicable to a completed psychiatry. These possibilities remain because Murphy’s arguments do not, explicitly at least, undermine the intelligibility of the concept of levels of explanation. By contrast, according to John Campbell, the very idea of a level of explanation is a reflection of a mistaken pre-Humean view of causation. There is less to explanation than the requirement to fit a specific level would require.

## Campbell’s Criticism of Levels of Explanation in Psychiatry

To characterize his target, Campbell gives the example of a discussion of thought insertion by Christopher Frith (9). Frith claims that whether or not inappropriate firings of dopamine neurons are found in subjects who experience thought insertion, this fact could not be used to explain their experiences. It would shed no light on why that kind of symptom, rather than another, was produced by inappropriate firings of dopamine neurons. To shed light, Frith assumes that we need an account pitched at a particular level: in Frith’s case that of a sub-personal but still cognitive model of mechanisms supposedly responsible for thought insertion.

Campbell suggests that the assumption that there is a right level of explanation that clarifies things in the way Frith desires is the result of a pre-Humean view of causal explanation. Although often forgotten, Hume successfully argued that there need be no intelligible connection between cause and effect. That is implicit in his rejection of any logical connection to analyze the apparently necessitating relation between cause and effect. Causal connections are merely brute facts to be discovered by experience.

Resisting the idea that the right kind of cause and effect have to be intelligibly, rather than merely brutely related also undercuts the motivation for the levels of explanation picture on both approaches: ontological and epistemic.

We naturally seek a certain kind of intelligibility in nature; we naturally try to find explanations that will show the world to conform to reason, to behave as it ought. Hume’s point is that there are no such intelligible connections to be found. This point has generally been accepted by philosophers thinking about causation. Hume’s comments nonetheless do leave us in an uncomfortable position, because we do tend to look for explanations that make the phenomena intelligible to reason. We are prone to relapse, to think that after all we must be able to find intelligibility in the world. This tendency survives, I suspect, in the idea of ‘levels of explanation’. The idea is that within certain levels of explanation, we will find a particular kind of intelligibility. [T]he lesson from Hume is that there is no more to causation than arbitrary connections between independent variables of cause and effect. We have to resist the demand for intelligibility [Ref. (2): p. 201].

This is not just a restatement of Murphy’s claim that, in psychiatry, explanations may cross levels. Rather, the very idea of levels of explanation, understood as causation operating under some constraint of intelligibility, is itself undercut. This applies to both ontological and epistemic versions as both assume that causation is governed by *a priori* constraints, whether degree of abstraction or composition.

This leaves, however, the issue of shedding some light on the nature of causal connections (if not *a priori* light on particular causal connections). In (non-mental) cases of causation, the notion of *mechanism* plays a central role in empirical research. Searching out the way in which causal influence is transmitted has been an important part of scientific practice. “It would seem a kind of madness if someone were to acknowledge that there is a causal link, but propose that there may be no mechanism linking the two” [Ref. (3): p. 138]. But if science has usefully explored the mechanisms that mediate causal influence, there must be some paradigmatic mechanisms that stand in need of no further explanation and the transmission of motion by impulse, in Hume’s billiard ball example, is one such prototype.

Nevertheless, the idea that there *must* be such a mechanism is a kind of synthetic *a priori* claim which, Campbell suggests, should be rejected in line with Hume’s argument. He adopts the interventionist model defended most extensively by James Woodward in *Making Things Happen* according to which for X to be a cause of Y is for intervening on X to be away of intervening on Y (10). The rejection of the necessity of a mechanism and the adoption of an interventionist approach opens up the possibility of a causal connection – in accord with interventionism – where there is no mechanism. In the case of psychiatry, however, the key issue is causation in the absence of a *mental* mechanism, whatever that is taken to be.

Just as we find it natural to expect there to be a mechanism underpinning material causal connections – even if this assumption lacks any genuine *a priori* justification – so Campbell also suggests that in the case of mental causation we expect there to be a rational connection between propositional attitudes. The rational link between two propositional attitudes is our paradigm of a mental causal mechanism. So, if one hears someone explain that they believe that Tranmere Rovers won their most recent football match because they heard it on the BBC, which they take to be trustworthy, no further inquiry is needed as to why the beliefs about what they hear and trust cause the belief about the result. Again, however, while the idea that mental causation is underpinned by rational connections is natural and compelling, it lacks *a priori* justification.

[T]here is an analogy between:

1 the idea that propositional attitude ascriptions depend on the ascription of rationality to the subject, and

2 the idea that all causal interactions between pieces of matter must be comprehensible in mechanistic terms.

Both ideas express an insight – that we find it extremely puzzling when we encounter causal relations among propositional attitudes that are not broadly rational, just as we find it extremely puzzling when we encounter causal interactions between physical objects that are not mechanistic, and that involve spooky ‘action-at-a-distance’. Both ideas express a natural impulse of philosophers – to elevate this kind of point into a kind of synthetic *a priori* demand that reason makes on the world. This impulse has to be resisted [Ref. (3): p. 142].

In both cases, there is a genuine insight. As a matter of custom and habit, we find an absence of material mechanisms and an absence of rational connections between mental states puzzling. But in both cases, it is a characteristic philosophical error to promote this natural expectation into a justified *a priori* claim that the world must respect. Mere custom and habit cannot rationally sustain any such demand on how the world must be.

The rejection of the necessity for rational connections between causally related mental states looks to ease a central problem for the philosophy of psychiatry: explaining delusions. There need be nothing genuinely mysterious about a causal connection, which lacks a rational connection (the expected mental mechanism).

Suppose you believe:

1 that this man is stroking his chin, and

2 that this man believes you need to shave.

What is it for the first belief to be a cause of the second? On the interventionist analysis, it is for the intervention on the first belief to be a way of changing whether you have the second belief. So if some external force changed your belief that this man is stroking his chin, you would no longer believe that he believes you need to shave. There is no appeal to rationality here, no appeals to mechanism [Ref. (3): p. 143].

The causal connection between one state and another is underpinned in interventionist terms based on the idea that if intervening on the first belief is a stable way of bringing about a change in the second then this is sufficient for there to be a causal connection between them.

Spelling this idea out involves a little more complexity, however. Given a scanner capable of yielding a complete microphysical description of the human body and a longitudinal study of schizophrenia in a population, Campbell suggests that it might be possible to form a disjunctive characterization of the set of microphysical states that are nomically sufficient for schizophrenia. But that function from physical states to illness would lack any concise expression and would not be couched in terms of variables, which could be affected by local intervention. This point reflects the pragmatic aspect to interventionism: not every nomically sufficient state counts as a cause.

For propositional attitudes to count as causes of delusions, Campbell suggests two conditions have to be met. There should be “systematic relations between cause variables and the subsequent delusion” and there should be a correlation between a change of the cause and a change of the effect [Ref. (3): p. 146]. More generally for the causal explanation of mental states, the causal variables, which he calls “control variables,” should have large, specific, and systematic correlations with their effects akin to the way the controls of a car systematically control its behavior. These conditions do not require a rational connection, however. To repeat Campbell’s phrase, there need be “no appeal to rationality here.”

The classical philosophical approach has been to regard propositional attitudes as part of a ‘conceptual scheme’ that we bring to bear in describing the ordinary world. This conceptual scheme is taken to have strong *a priori* constraints on its applicability. In particular, as we have seen, rationality is taken to be a norm with which the scheme has to comply. The appeal I have just been making to the notion of a control variable is intended to replace this invocation of rationality. [I]t is the fact that we have control variables, not the fact that we have rationality, which means that we are ‘at the right level’ to talk of beliefs and desires [Ref. (3): p. 147].

The phrase “at the right level” occurs in inverted commas to flag the fact that the notion of the right explanatory level has been undercut. Without a pre-Humean insistence on the intelligibility of causal relations, there is no more to the notion of being at the right level than that there is a causal relation tracked through the idea of control variables.

With the idea of control variables replacing an *a priori* requirement for rationality in mental causation, psychiatric explanation of delusions is in principle in the same predicament as the explanation of any other belief. Causal explanation has been achieved once one has an understanding of the variables necessary for changing the delusional belief entertained. The apparently principled problem of attempting to fit primary delusions into some sort of rational framework is replaced by a practical problem of charting the variables that affect them. But is that minimal approach enough for psychiatric explanation?

## Wittgenstein on Causation and Mechanism

Campbell suggests a mutually supportive analogy between the denial that mental causation requires rational mediation and that physical causation requires a mechanism. The latter denial echoes some remarks by Wittgenstein in *Zettel*. In this section, I will outline the context of Wittgenstein’s discussion, outline a key disanalogy and hence begin to suggest a reason to reject explanatory minimalism in psychiatry.

The later Wittgenstein makes a number of comments both explicitly and implicitly on the connection between mind and body. Throughout his various discussions of propositional attitudes, he denies the possibility of an explanation of meaning or forming an intentional mental state via an appeal to brain states. This accords with his criticisms of causal and dispositional explanations of rule following in the *Philosophical Investigations* (11). As the discussions of both real and ideal machines imply, the attempt to explain rules by appeal to mechanisms is either question begging or fails to sustain their normativity [ibid §§193–4]. Thus, no account could be given in which thought processes might be read off from brain processes.

Such considerations might be thought to motivate the following claim in *Zettel*:

No supposition seems to me more natural than that there is no process in the brain correlated with associating or with thinking; so that it would be impossible to read off thought-processes from brain-processes. I mean this: if I talk or write there is, I assume, a system of impulses going out from my brain and correlated with my spoken or written thoughts. But why should the *system* continue further in the direction of the centre? Why should this order not proceed, so to speak, out of chaos? [Ref. (5): §608]

It is thus perfectly possible that certain psychological phenomena *cannot* be investigated physiologically, because physiologically nothing corresponds to them [ibid: §609].

These passages could be interpreted as merely denying that the systematicity of thought can be explained as resulting from an underlying systematicity in the brain. In other words, they could be interpreted as a denial of reductionist explanations of meaning and mental content.

This interpretation would also be consistent with another passage:

Imagine the following phenomenon. If I want someone to take note of a text that I recite to him, so that he can repeat it to me later, I have to give him paper and pencil; while I am speaking he makes lines, marks, on the paper; if he has to reproduce the text later he follows those marks with his eyes and recites the text. But I assume that what he has jotted down is not *writing*, it is not connected by rules with the words of the text; yet without those jottings he is unable to reproduce the text; and if anything in it is altered, if part of it is destroyed, he sticks in his ‘reading’ or recites the text uncertainly or carelessly, or cannot find the words at all. – This *can* be imagined! – What I called jottings would not be a *rendering* of the text, not so to speak a translation with another symbolism. The text would not be *stored up* in the jottings. And why should it be stored up in our nervous system? [ibid: §612]

This passage does not say that the marks on paper do not form a system. It is just that they do not form a system of the same sort as writing. That is why they are not a *rendering* of the text. They are not connected by *rules* to words. But in that case, what is their connection to the text supposed to be? Given that this is supposed to be an analogy for the connection between the nervous system and our linguistic abilities, one suggestion is that the marks are connected to written or spoken words *causally* rather than via shared meaning. If this were the case, while the internal system could not be used to *reduce* mental content, it could still play a necessary causal role.

But in fact, Wittgenstein goes further than this. He suggests that there need be *no* cause of a memory in the nervous system. Nothing need be stored “up there” in any form. There need be no physiological regularity or order causing psychological order. Mental order could proceed out of chaos:

The case would be like the following – certain kinds of plants multiply by seed, so that a seed always produces a plant of the same kind as that from which it was produced – but *nothing* in the seed corresponds to the plant which comes from it; so that it is impossible to infer the properties or structure of the plant from those of the seed that it comes out of – this can only be done from the *history* of the seed. So an organism might come into being even out of something quite amorphous, as it were causelessly; and there is no reason why this should not really hold for our thoughts, and hence for our talking and writing [ibid: §608].

I saw this man years ago: now I have seen him again, I recognise him, I remember his name. And why does there have to be a cause of this remembering in my nervous system? Why must something or other, whatever it may be, be stored up there *in any form*? Why *must* a trace have been left behind? Why should there not be a psychological regularity to which *no* physiological regularity corresponds? If this upsets our concepts of causality then it is high time they were upset [ibid: §610].

These two passages are pitched against a model of levels of explanation in which a psychological regularity corresponds to a regularity at a lower level. That is, they run counter to the assumption in both Putnam and Oppenheim’s ontological, but also Marr’s epistemological, accounts of the constraints operating between levels of explanation. By contrast with Campbell, Wittgenstein does not reject the idea that there is a characteristic level of explanation for mental happenings. Using terminology not in widespread use when Wittgenstein wrote these remarks, they amount to the claim that plants’ development do not supervene on their seeds’ microstructure. Given contemporary understanding of RNA, this might seem a bizarre possibility. But the denial is of a *modal* claim: that the plant *must* be determined by something in the seed’s structure. Wittgenstein denies this assumption, natural though it currently seems.

Like Campbell, Wittgenstein denies that the psychological regularity has to be mediated by one at the neurological or physical level. Furthermore, like Campbell, he suggests an analogical denial. What we might have taken to be a causal physical connection – in the seed and tree example – also need not be mediated by any mechanism. (The analogy is with psychological regularity depending on the physical level.) There is a difference between Wittgenstein and Campbell, however, in that Wittgenstein here assumes the very connection between causation and mechanism that Campbell denies in favor of interventionism. Wittgenstein asks:

Why should there not be a natural law connecting a starting and a finishing state of a system, but not covering the intermediary state? (Only one must not think of *causal efficacy*.) [ibid: §613]

This passage assumes that the denial of an intervening mechanism implies a denial of causal efficacy. Given that the natural law would sustain the kind of intervention conditionals, then a particular kind of seed producing a particular kind of plant would count as a causal connection according to Campbell. But this looks merely like a difference of terminology. Both Campbell and Wittgenstein can grant a law-like connection. Campbell’s account suggests it should count as “causal” while Wittgenstein denies it “causal efficacy.” Both deny that there need be an intermediate mechanism. But aside from its causal status, the metaphysical facts are agreed.

Despite that, however, there is a fundamental difference. Wittgenstein’s remarks are aimed at removing the tension of reconciling a connection made at the mental level in mental and, according to him, non-causal terms with assumptions about underlying causal mechanisms at a physiological level. Campbell’s, by contrast, suggest that transitions at the mental level can, when explanatory, be causal. I will now explore the significance of this difference.

## The Role of an Appeal to Rationality in Wittgenstein’s Discussion of Intentionality

In order to see the difference between Campbell and Wittgenstein, it will be helpful to start with what they share. There are two particularly clear examples in the *Philosophical Investigations* where Wittgenstein, like Campbell, rejects an appeal to underlying mechanisms to explain a connection at the mental level. But, by contrast with Campbell, he goes on to suggest a different account of the mental connection. It is this that suggests Wittgenstein’s commitment to a characteristically mental level of explanation.

One example concerns the ability to read out loud, of what reading comprises. He considers the temptation to identify the ability with a mechanism through the example of a comparison between an expert reader and a beginner who can only read words by laboriously spelling them out.

Now we would, of course, like to say: What goes on in the practised reader and in the beginner when they utter the word *can’t* be the same. And if there is no difference in what they are currently conscious of, there must be one in the unconscious workings of their minds, or, again, in the brain. – So we’d like to say: There are, at any rate, two different mechanisms here! And what goes on in them must distinguish reading from not reading. – But these mechanisms are only hypotheses, models to explain, to sum up, what you observe [Ref. (11): p. §156].

Rejecting the hypothetical mechanism – whether an unconscious mental mechanism or physiological one – as well as conscious experiences of being guided or feelings of familiarity, he stresses instead the relation between the text and spoken words, however, mediated. Whatever mediating processes there may be are not what is meant by “reading.”

A second example concerns the intentional directedness of having someone in mind.

“I am thinking of N.” “I am speaking of N.”

How do I speak *of* him? I say, for instance, “I must go and see N. today” – But surely that is not enough! After all, when I say “N.”, I might mean various people of this name. – “Then there must surely be a further link between my words and N., for otherwise I would *still* not have meant him.” Certainly such a link exists. Only not as you imagine it: namely, by means of a mental *mechanism* [ibid: §689].

In the surrounding discussion, various putative explanatory connections are considered and rejected including the idea that no such connection exists, that it is created in being verbally avowed (and that both are true!), and that it is connected to what would, counter-factually, have been reported. Wittgenstein’s discussion fits a meta-philosophical injunction: “The point is not to explain a language-game by means of our experiences, but to take account of a language-game” [ibid: §655]. But it also accords with a brief assertion in the middle of an earlier discussion of the intentional directedness of propositional attitudes: “It is in language that an expectation and its fulfilment make contact” [ibid: §445].

This terse comment picks up the idea that avowals and descriptions of expectations and other propositional attitudes reuse the same fragments of language as descriptions of the events that would satisfy them (12). To be able to form such a propositional attitude requires the contingent ability to fit one’s avowals and actions into the rational pattern articulated in language. The criticism of underlying mechanisms is made against the background account that psychological order has a rational linguistically mediated structure.

This suggests a fundamental contrast with Campbell’s view. Although both Campbell and Wittgenstein reject mechanisms, Wittgenstein’s rejection goes hand in hand with a normative and rationalistic view at the mental level which Campbell, at least in the series of papers so far discussed, downplays. In the final section, I will outline the consequences of this disagreement for causal explanation in psychiatry. But first I will briefly summarize how Wittgenstein’s views of meaning and mental content suggest a picture of levels of explanation.

## A Wittgensteinian View of Levels of Explanation

I began by outlining the two dominant approaches to thinking about levels of explanation, both ontological and epistemic. Both approaches not only suggest ways of distinguishing levels but both also suggest constraining relations of either composition, in the ontological case, or realization, in the epistemological approach. Wittgenstein’s discussion of mental phenomena in, especially, his *Philosophical Investigations* sets out some of the key differences between normative meaning-related or intentional connections and causal connections. But his remarks in *Zettel* run counter to the assumptions, particularly in Putnam and Oppenheim, of the constraining relations between the psychological and the neurological.

In other words, Wittgenstein’s remarks suggest a middle ground between Campbell, on the one hand, and Putnam and Oppenheim, on the other hand. Thinking that there are distinct forms of intelligibility need not imply an *a priori* view of a constraining relation between them. Putnam and Oppenheim assume a series of levels of explanation but then impose a reductionist view of their relations. Campbell rejects the intelligibility of levels of explanation in the first place. Wittgenstein, however, suggests that grasping events or states as mental phenomena presupposes fitting them into a normative and rational linguistic structure but denies that this necessitates connections to a non-normative pattern of causal relations. This suggests that to understand a state to be a state of expectation, for example, involves relating it in a characteristic way to events that would satisfy or fulfill it and hence to presuppose a particular *a priori* pattern of intelligibility. But Wittgenstein denies the need, *a priori* at least, to connect this to any underlying pattern of neurological cause and effect.

The denial of an *a priori* connection to underlying neurology is not the same as denying an *a posteriori* connection. The remarks in *Zettel* do not contradict the possibility of neurological and psychiatric research establishing local connections between medical interventions and psychological effects. Instead, they caution merely against assuming that a pattern at one level must be relatable to a pattern at a lower level.

## Inflating Explanatory Minimalism in Psychiatry

Earlier I reported Campbell’s claim that:

[I]t is the fact that we have control variables, not the fact that we have rationality, which means that we are ‘at the right level’ to talk of beliefs and desires [Ref. (3): p. 147].

I asked whether the resulting picture of explanatory minimalism was sufficient for psychiatric explanation. It is not sufficient because it provides no account of what constitutes a state as a belief, or a desire or even a delusion. In the absence of that, however, psychiatric explanation would miss a key feature of the phenomena it aims to illuminate.

In an earlier paper, Campbell himself endorses the role of rationality as a presupposition for holding propositional attitudes. He suggests two general reasons for this. The less important one is as follows.

One simple reason for thinking that rationality is critical here is that unless you assume the other person is rational, it does not seem possible to say what the significance is of ascribing any particular propositional state to the subject. If you tell me that someone rational thinks that it is raining, then given that the person is rational and does not want to get wet, I know what kinds of behavior to expect. If, however, the person is not at all rational, then saying they have the belief has no implications at all for how they will behave [Ref. (13): p. 89].

Campbell’s focus is on the *ascription* of propositional attitudes to others. The imputation of rationality goes hand-in-hand with an ascription of propositional attitudes. The argument in the passage seems to concern what follows from the ascription. Without the assumption that the subject is also rational, it is not clear what can be inferred from the ascription of particular mental states to them. But this argument surely broadens. Without a rational pattern, the very idea that the subject has some determinate mental state is undermined (14–16).

There is a second connection, however, which Campbell thinks is the more important. It concerns the connection between rationality, belief, and meaning. Understanding others’ utterances and hence ascribing beliefs to them is only possible against a background assumption of rationality. There is a balance between possible irrationality and the ascription of meaning.

The finding of irrationality can always be traded for a finding of mistranslation. And we should always translate so as to find the subject rational in the use of a term by the lights of the subject’s own understanding of the term [ibid: 90].

This sketch of the connection between interpretation and the ascription of belief echoes Wittgenstein’s suggestion of a linguistic mediation of mental states and their intentional objects. The very idea of having propositional attitudes presupposes a harmony between the meaning of utterances, the mental states held, and the pattern of actions they rationalize.

Thus, the claim that control variables, rather than rationality, constitutes the “right level” to talk of beliefs and desires fails to address a prior constitutive question. What is it about some particular causes and effects, described using the interventionist model of causation, which constitutes them as intentional mental states in the first place? Given its broad application to causation in the non-mental as well as mental world, talk of control variables alone is insufficient to address this question. But introducing issues of language, interpretation, and rationality suggests that there is a particular level of explanation which is of central importance to psychiatry when it addresses the meaning and content of psychological phenomena.

In the example described above, Campbell argues that for the belief that this man is stroking his chin to cause the belief that this man believes you need to shave all that is needed is a suitable interventionist counterfactual relation rather than an appeal to rationality. But without some further background conditions, of which rationality is one plausible candidate, the ascription of determinate mental states is illicit.

It may seem, however, that defending the role of a rational connection between utterance, mental state, and behavior is particularly difficult in the case of psychiatric explanation. After all, psychiatry investigates phenomena that appear to resist rational understanding. While this is true and puzzling, however, it does not threaten the connection itself.

Consider Campbell’s discussion of Capgras in the earlier paper (13). He uses the link between meaning and rationality to suggest a problem with the interpretation of characteristic expressions of the delusion. The characteristic type of utterance associated with the delusion is: “That woman is not my wife!.” But that sentence might be used to make a number of different claims. It might, for example, be used to flag the discovery of illegality in a past wedding ceremony. The most plausible interpretation in the context of the expression of the Capgras delusion is something like: This [demonstrated] woman is not that [remembered] woman. But such an interpretation is put under strain because, typically, the subject of the delusion does not attempt to carry out any of the paradigmatic or canonical forms of checking appropriate for such a claim: for example, discussing past events and checking memories. They do not do what they ought to do to check such a thought. Given the link between meaning, mental content, and rationality, this apparent failure of rationality undermines such an interpretation.

Campbell himself goes on to try a partial accommodation of the delusion within rational space by suggesting it might be a deviant hinge or framework proposition since, if it were, it would be rational not to subject it to testing. It is unclear whether this approach can work as it is unclear what understanding there can be of a framework proposition which is not shared (17). But the difficulties Campbell highlights seem genuine. Does a subject who makes a paradigmatic Capgras utterance but does nothing else different really believe that their partner is an imposter? Likewise, does the Cotard utterance “I am dead” really express the impossible belief that the subject is dead? The difficulty seems fundamental to such cases.

In a more recent paper, Campbell seems more pessimistic about fitting delusions into any sort of rational pattern. He considers a delusion in which the subject thinks that her mother’s thoughts were inserted into her mind via raindrops and the air conditioner. He points out that the structure of this delusion could not be used to teach what is meant by “rationality.” But further:

The trouble is not even that the patient is not rational. We have no idea what a rational way of going on would be, once one has accepted that thoughts are being inserted into one’s mind. How must the world be, for that to happen? Would it make sense to argue with this patient that, by her own lights, it is not the raindrops in the air conditioning that should be blamed, but rather the electrical sockets all around? We have departed so far from the ordinary world that we have no idea what stands fast and what has to go [Ref. (3): p. 141].

Again, these seem to be genuine and substantial difficulties in working out what the subject actually thinks. But Campbell offers a particular interpretation of the difficulty. He says:

We should not appeal to the idea that there are *a priori* constraints on causal relations among propositional attitudes. We have to accept that the propositional attitudes are one thing and the causal relations among them are another. If propositional attitudes do not conform to rationality, that is puzzling. But we cannot legislate in advance that this cannot happen [ibid: 140].

This seems an unjustified response, however. The problem is not merely that there are contingent breakdowns in the expected rational connections between identifiable propositional attitudes. Rather, in the case of delusion, the nature of the supposed propositional attitudes themselves is, and continues to be, puzzling. Hence, for example, attempts to suggest that the delusion may be a propositional attitude of imagination rather than belief [e.g., Ref. (18)]. It is not that the bizarre quality of delusions threatens the general connection between meaning, mental state, and rationality but instead that the general connection helps to illuminate what is so puzzling about delusion. The connection to rationality is not arbitrary: it helps justify the claim that a state is a mental state or that an utterance expresses a particular propositional attitude.

## Conclusion

Given the heterogeneity of the factors that feature in explanations in psychiatry, it is tempting to assume that, in principle, they can be related within an ordered hierarchy of levels of explanation. There is reason, however, to doubt that this picture fits contemporary psychiatry. But that leaves open the response that that is a reflection merely on the current state of psychiatric research and that a completed psychiatry would form an ordered hierarchy.

More radically, John Campbell has argued in recent papers that the very idea of levels of explanation presupposes a discredited pre-Humean view of causation. He claims that although the assumption that physical causation is mediated by mechanisms and that psychological causation is mediated by rational relations have both been fruitful neither need to be true. With their rejection as synthetic *a priori* claims about the world, the idea of levels of explanation also falls away to leave an explanatory minimalism.

Comparing Campbell’s remarks with some superficially similar remarks in Wittgenstein’s *Zettel* suggests an objection to explanatory minimalism. The very idea of a state being a mental state presupposes broader connections. Rationality is one such candidate. If so, explanation in psychiatry inflates from Campbell’s minimalism and introduces an appropriate level of explanation at which mentality comes into view. But it is possible to hold on to the necessity of such general levels of explanation while rejecting *a priori* claims about how different levels of explanation must relate to each other.

## Conflict of Interest Statement

The author declares that the research was conducted in the absence of any commercial or financial relationships that could be construed as a potential conflict of interest.
